# 

**Published:** 1904-06

**Authors:** 


ESTABLISHED IN 1839

THE OLDEST DENTAL MAGAZINE IN THE WORLD

INDEPENDENT---------PUBLISHED BY DENTISTS FOR DENTISTS

VOLUME XXXV NUMBER 6 | THIRD SERIES | JUNE 1904

EDITOR:
WM. GIRD BEECROFT, D. D. S., Madison, Wis.
ASSOCIATE EDITORS:
ALBERT H. KETCHAM, D. D. S., Denver, Colo.
EMORY A. BRYANT, D. D. S., Washington, D. C.
GEO. S MARTIN, D. D. S., L. D. S ,Toronto Junction, Ontario.
Publisheu on the 10th day of each month.
Subscription Price, $1.00 per year.
Foreign Countries, $1.50 pe: year.
Address all communications, both business and editorial to the editor.
**Entered as Second Class Matter, Madison, Wis.**

